# The radiosensitizer 2-benzoyl-3-phenyl-6,7-dichloroquinoxaline 1,4-dioxide induces DNA damage in EMT-6 mammary carcinoma cells

**DOI:** 10.1186/1748-717X-4-25

**Published:** 2009-07-14

**Authors:** Joelle Haykal, Fady Geara, Makhluf J Haddadin, Colin A Smith, Hala Gali-Muhtasib

**Affiliations:** 1 Department of Biology, American University of Beirut, Beirut, Lebanon; 2 Department of Radiation Oncology, American University of Beirut, Beirut, Lebanon; 3 Department of Chemistry, American University of Beirut, Beirut, Lebanon

## Abstract

**Background:**

DCQ (2-benzoyl-3-phenyl-6,7-dichloroquinoxaline 1,4-dioxide), a synthetic quinoxaline 1,4-dioxide, enhances the cytotoxic effect of ionizing radiation (IR) *in vivo *and *in vitro*. We sought to clarify whether increased radiation-induced DNA damage, decreased rate of damage repair, and the generation of reactive oxygen species (ROS) contribute to DCQ enhancement of IR.

**Methods:**

Murine mammary adenocarcinoma EMT-6 cells were treated with DCQ for 4 h before exposure to 10 Gy IR. Treated cells were monitored for modulations in cell cycle, induction of DNA damage, and generation of ROS.

**Results:**

Combined DCQ and IR treatments (DCQ+IR) induced rapid cell-cycle arrests in EMT-6 cells, particularly in S and G_2_/M phases. Alkaline comet assays revealed high levels of DNA damage in cells after exposure to DCQ+IR, consistent with damage-induced arrest. Unlike IR-only and DCQ-only treated cells, the damage induced by combined DCQ+IR was repaired at a slower rate. Combined treatment, compared to separate DCQ and IR treatments, activated DNA-protein kinase and induced more p-ATM, supporting a role for double strand breaks (DSBs), which are more toxic and difficult to repair than single strand breaks (SSBs). Contributing factors to DCQ radiosensitization appear to be the induction of ROS and DSBs.

**Conclusion:**

Collectively, our findings indicate that radiosensitization by DCQ is mediated by DNA damage and decreased repair and that ROS are at least partially responsible.

## Background

Eukaryotic cells have evolved DNA damage checkpoints that control the fate of an insulted cell by inducing cell-cycle arrest, repair of the damage, and cell death. Many malignant cells have incompetent cell-cycle controls, and hence, DNA synthesis and replication may proceed despite the presence of unrepaired DNA damage, leading eventually to unviable daughter cells [1,2]. Thus, such malignant cells are sensitive to therapies that induce DNA damage [3].

Some aromatic N-oxides such as quinoxalines induce DNA damage in cancer cells. The hypoxic cytotoxin 7-chloro-3-[(N, N-dimethylamino) propyl]amino]-2-quinoxalinecarbonitrile 1,4-dioxide hydrochloride (Q-85 HCl) has been shown to induce DNA damage under hypoxic conditions in CaCo-2 cells by producing reactive oxygen species (ROS) [4,5]. The mechanism of action of such compounds is not yet clear. However, studies on quinoxaline 1,4-dioxide has shown that it is reduced enzymatically into an active, oxygen-sensitive radical responsible for DNA cleavage [6].

A similar quinoxaline, 2-benzoyl-3-phenyl 6,7-dichloroquinoxaline 1,4-dioxide (DCQ) has been shown to be cytotoxic and a radiosensitizer on several cancer cell lines, including colon cancer cells. The radiosensitization effect was also shown *in vivo*, using C57BL/6 mouse model [7]. Combined treatment with DCQ and radiation delayed the growth of LLC tumors injected in the mice and reduced the mean tumor volume by 80% [7]. Recent results have shown that DCQ causes DNA damage in DLD-1 colon cancer cells [8]. Despite data *in vitro *and *in vivo *confirming that DCQ is a radiosensitizer little is known about its mechanism of action. In this study, we first assessed the effects of DCQ ± IR on cell cycle progression at early time-points. Then, we tested whether DCQ radiosensitization is associated with an enhancement in radiation-induced DNA damage or with a decrease in the rate of damage repair. Finally, we investigated the possible involvement of ROS in the mechanism of DCQ toxicity.

## Methods

### Chemicals

RPMI 1640 with 25 mm HEPES and L-glutamine, Dulbecco's modified eagle medium nutrient mixture F12, fetal bovine serum, trypsin, penicillin-streptomycin and Dulbecco's Phosphate Buffered Saline (PBS) were purchased from Gibco BRL Life Technologies (Gaithersburg, Maryland, US). The Cytotox non-radioactive cytotoxicity assay kit and the Cell Titer 96 non-radioactive cell proliferation assay kit were purchased from Promega Corp (Madison, Wisconsin, US). Propidium iodide (PI), YOYO-1 dye, fluorescein isothiocyanate (FITC) goat anti-mouse IgG (H+L), and 5-(and-6)-chloromethyl-2',7'-dichlordihydrofluorescein diacetate, acetyl ester (CM-H_2_DCFDA) were purchased from Molecular Probes (Eugene, Oregon, US). RNase A, dimethylsulfoxide (DMSO) and N-acetyl cysteine (NAC) were obtained from Sigma Chemical Company (St. Louis, Missouri, US). ATM kinase phosphoser1981 antibody was obtained from Chemicon International (California, US). DCQ was synthesized from 5,6-dichlorobenzofurazan oxide and dibenzoylmethane by the Beirut Reaction [9].

### Cell Culture, Drug and Irradiation Treatment

The murine mammary adenocarcinoma cell line EMT-6 was cultured in growth media containing RPMI 1640 with L-glutamine and 25 mm HEPES, supplemented with 10% FBS and 1% penicillin-streptomycin (50 μg/mL), and incubated in a humidified incubator (95% air 5% CO_2_) at 37°C (Forma Scientific Inc. Ohio, US).

DCQ was dissolved in DMSO at a concentration of 10 mg/mL. Prior to treatment, it was diluted in media containing FBS. EMT-6 cells were plated at a density of 16 × 10^3 ^cells/cm^2^. At 50% confluency, they were incubated with DCQ (0–10 μM) for 4 h prior to irradiation (0–10 Gy).

Cells were irradiated at room temperature using a high dose rate Cesium-137 Laboratory Irradiator (JL Shepherd) that delivers gamma-irradiation at a dose rate of 174 cGy/min. After irradiation, cells were replenished with fresh media containing no drug and incubated for different times.

The murine mammary epithelial cell line SCp2 (kindly provided by R. Talhouk, Biology Department, American University of Beirut, Lebanon) was used as a model for normal, slowly proliferating cells [10]. SCp2 cells were grown in normal growth media composed of DMEM: F12 supplemented with 5% FBS, 1% Penicillin-Streptomycin, and 0.1% insulin (5 μg/mL, Sigma, St. Louis). To induce differentiation of the SCp2 cells, the cells were plated in growth media and 12 later the media was replaced with differentiation medium lacking FBS [10]. The differentiation medium consisted of DMEM: F12 supplemented with 0.1% insulin (5 μg/mL), 0.1% hydrocortisone (1 μg/mL), and 0.1% prolactin (3 μg/mL). For a more differentiated state, a growth factor reduced basement membrane derived from Engelbreth-Holm-Swarm tumor was added 12 h after plating. A basement membrane is known to induce differentiation in SCp2 cells by making their environment more similar to that of normal cells [10].

### Proliferation and Cytotoxicity Assay

Cells were plated at a density of 10^5 ^cells/mL in 96-well plates. After 24 h, cells were treated in triplicates with different DCQ concentrations. In some experiments, EMT-6 cells were pre-treated with either NAC (5 mM) or Tiron (1 mM) for 2 h prior to DCQ treatment.

Cytotoxicity was performed after 4 h of DCQ treatment using the Cell Titer 96 non-radioactive cytotoxicity kit. Briefly, supernatants were mixed with a substrate mix containing tetrazolium salt that interacts with lactate dehydrogenase, a stable cytosolic enzyme that is released into the supernatant upon cell lysis. The interaction results in the conversion of the tetrazolium salt into a red formazan product, the absorbance of which is recorded at 492 nm.

As for the proliferation assays, cells were replenished with drug-free media after the 4 h-DCQ treatment, and were incubated for 20 h before the assay was performed using the Cell Titer 96 non-radioactive cell proliferation. This assay measures the ability of metabolically-active cells to convert tetrazolium salt into a blue formazan product that can be measured by its absorbance at 595 nm.

### Flow Cytometry

Cells were either treated with 0.1% DMSO (control), DCQ (0–10 μM) for 4 h, irradiation (10 Gy), or combinations. Immediately after radiation or drug treatment, cells were replenished with fresh media containing no drug and incubated for 0 h, 2 h, and 4 h. Subsequently, cells were harvested and fixed in ice-cold 70% ethanol and stored at -20°C. On the day of DNA staining, cells were incubated in 0.2 mg/mL RNase A at 37°C, and stained with 6.25 μg/mL PI for 30 min in the dark at room temperature. Finally, cell cycle analysis was performed using a Fluorescence Activated Cell Sorter (Becton Dickinson, Research Triangle, NC), and the percentages of cells in sub-G_1 _(< 2n), G_0_/G_1_, S and G_2_/M phases were determined using the Cell Quest program (BD Biosciences, California, USA).

### DNA Damage Detection by the Alkaline Comet Assay

The alkaline comet assay used is a modification of the method developed by Singh that detects the frequency of SSBs and alkaline-labile lesions in DNA [11]. Microscope slides were coated with 1% normal melting agarose, and left overnight to dry. Cells suspended in media were mixed with 75 μL of 0.5% low-melting-point agarose (LMPA) and were distributed on the coated slide. The slides were left to gel for 10 min at 4°C, before a third layer of 80 μL 0.5% LMPA was added to the slide and left for 10 min at 4°C. The slides were then dipped in cold lysing solution (1.25 M NaCl, 50 mM EDTA, 100 mM Tris base and 0.01% sodium lauroyl sarcosine; pH 10) for a minimum of 2 h at 4°C. Before proceeding, the slides were incubated in pre-warmed lysing buffer containing DNAse-free proteinase K for 1 h at 37°C. The slides were transferred to an electrophoresis unit filled with electrophoresis buffer (300 mM NaOH, 1 mM EDTA, 0.2% DMSO, and 0.1% 8-hydroxyquinoline; pH ~12.3), and were left immersed in the solution for 20 min, before being subjected to electrophoresis. Electrophoresis was carried out for 20 min at a voltage of 0.5 V/cm and a current of 250 mA. Next, the slides were rinsed with neutralization buffer (20 mM Tris, 1 mg/mL spermine, and 50% ethanol; pH 7.4) for 10 min. Finally, each slide was stained with 50 μL of YOYO-1 stain (0.25 μM YOYO-1, 2.5% DMSO and 0.5% sucrose). YOYO-stained nuclei were observed and photographed using a fluorescence microscope (AXIOVERT 200, ZEISS Fluorescence and optical microscope with ZEISS AXIOCAM HRC (Germany) and KS 300 V3 image analysis software) illuminated with blue light (490 nm). Images of a minimum of 50 cells per treatment were analyzed using the CometScore™ software. In the present study, percentage of DNA in the tail region, and tail moment (%DNA in tail × by tail length (μm)) were used as parameters to assess DNA damage.

### Immunocytochemistry Detected by Flow Cytometry

Ser-1981-phosphorylated ATM (p-ATM) was detected immunocytochemically by multiparameter cytometry with respect to the cell cycle phases, using the method developed by Huang and Darzynkiewicz [12]. Cells were collected by trypsinization, centrifuged, washed with PBS, and fixed with ice-cold 70% ethanol for a minimum of 2 h at -20°C. Ethanol was discarded by centrifugation at a speed of 10000 rpm for 5 min, and the pellets were washed with BSA-T-PBS containing 1% BSA and 0.2% Triton X-100 dissolved in PBS. The pellets were blocked in BSA-T-PBS for 5 min at room temperature. After removal of the 1% BSA solution by centrifugation, the cells were incubated with the primary antibody Ser-1981-p-ATM at a dilution of 1:100 overnight at 4°C. The cells were washed twice with BSA-T-PBS, and the pellets were then incubated in the dark with fluorescein isothiocyanate (FITC)-conjugated secondary anti-mouse antibody (1:30) for 1 h at room temperature. A volume of 5 mL of BSA-T-PBS was added to the cell suspension and kept for 2 min before centrifugation at 12000 rpm for 4 min. Finally, the cells were counterstained with PI (5 μg/mL) solution containing RNase A (0.1 mg/mL) for 30 min at room temperature in the dark. Both the fluorescence of PI and FITC of 10^4 ^cells/treatment were measured using the FACS cytometer, and analyzed using Cell Quest.

### Detection of ROS by DCFDA assay

Cells were plated at a density of 16 × 10^3 ^cells/cm^2 ^and treated at 50% confluency with 10 μM DCQ for 30 min. Control and treated cells were collected by trypsinization, centrifuged, washed with PBS, and incubated in 500 μl of media (with 2% FBS) containing 10 μM of DCFDA for 20–30 min at 37°C. DCFDA is a chemically-stable, non-fluorescent molecule that is hydrolyzed to DCFH inside the cell. DCFH interacts with ROS to form a fluorescent complex. Samples were then centrifuged, washed with PBS, and then resuspended in 500 μl of PBS. The fluorescence of DCF was immediately measured by flow cytometry.

### Chromatin Immunoprecipitation Followed by Western Blot

Chromatin immunoprecipitaion was performed using Chromatin Immunoprecipitation (CHIP) Assay Kit (Upstate, New York, USA) according to the manufacturer's protocol. Briefly, EMT-6 cells were treated at 70% confluency. DNA-binding proteins were cross-linked to DNA by adding 1% formaldehyde for 10 min at 37°C. Cells were washed twice with ice-cold PBS containing protease inhibitors (1 mM PMSF, 1 μg/mL aprotinin and 1 μg/mL pepstatin A). Cells were collected and centrifuged at a speed of 2000 rpm for 4 min at 4°C. Pellets (of 10^6 ^cells) were lysed with SDS Lysis buffer (provided by the kit) containing protease inhibitors. The chromatin, including bound proteins, was sonicated into smaller fragments (200–1000 base pairs) using Misonix Sonicator 3000 at 10% power (3 W) for seven 10-second pulses separated by a 5 second-pause. Samples were centrifuged for 10 min at 13000 rpm at 4°C, and the supernatants were diluted 10 fold in CHIP dilution buffer (provided by the kit) and pre-cleared with protein A agarose/salmon sperm DNA (50% slurry). DNA-PK antibody (1.5 mg/mL) was used to co-immunoprecipitate the protein-DNA complex which was then washed with different buffers: low salt immune complex, high salt immune complex, LiCl immune complex wash buffers, as well as two washes with TE (Tris-EDTA) buffer. Proteins were dissolved in 25 μL 1× sample buffer, boiled for 10 min, and resolved on a 5% acrylamide gel to detect the level of DNA-PK by western blotting.

### Western Blot

Proteins were resolved by sodium dodecyl sulfate-polyacrylamide gel electrophoresis (SDS-PAGE) on a 5% polyacrylamide gel, and transferred onto an activated polyvinylidene difluoride (PVDF) membrane in cold transfer buffer (14.4 g of glycine, 3 g Tris base, and 1 g SDS dissolved in 1 L of 20% methanol) at 30 volts overnight. The membrane was then blocked for 1 h with 5% non-fat milk dissolved in Tris-buffered saline (TBS) containing 0.1% Tween-20, and probed with DNA-PK antibody diluted in 1% blocking buffer overnight at 4°C. The membrane was incubated with horseradish peroxidase-conjugated secondary antibody 1 h at room temperature. The membrane was exposed to X-ray film (Hyperfilm ECL) using chemiluminescent substrate (Amersham).

## Results

### DCQ Induces S Phase and G2/M Arrest in EMT-6 Cells

**Figure 1:**
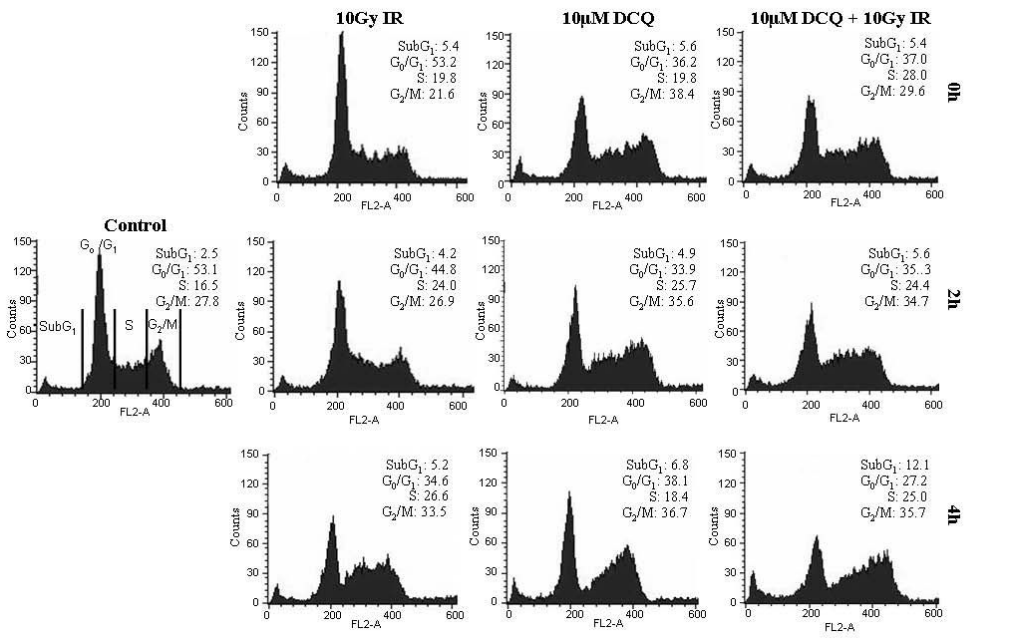
**S and G2/M arrest induced by DCQ ± IR in EMT-6 cells at 0, 2, and 4 h post-treatment**. EMT-6 cells were treated at 50% confluency with DCQ (10 μM) for 4 h, and/or irradiated (10 Gy). Immediately after the 4 h-drug incubation or IR exposure, cells were replenished with media containing no drug. Cells were collected at 0 h, 2 h, and 4 h after the refreshment of media and subjected to flow cytometry. Percentages of cells in the sub-G1 (A), S (B), and G2/M (C) phases of the cell cycle were determined by CellQuest and the averages ± SD are plotted for each treatment. Dashed lines represent the % of control cells in each phase of the cell cycle. SD: standard deviation.

Previous work has shown that DCQ, in combination with IR, induces apoptosis in EMT-6 cells 24 h post-treatment, and decreases their clonogenic survival [13]. To determine the direct effects of DCQ ± IR on cell cycle progression of EMT-6, cells were treated with 10 μM DCQ for 4 h followed by irradiation with 10 Gy IR, or separately treated. Treated cells were collected for flow cytometry either directly (0 h), or at 2 h or 4 h after IR exposure (Figure 1). IR induced cell-cycle arrest in the S phase at 2 h post-exposure and this arrest increased at 4 h with 27% of the population accumulated in the S phase. DCQ alone also caused an accumulation of cells in the late S phase immediately after drug treatment, and this accumulation increased to 26% after 2 h. Although IR alone and DCQ alone caused similar level of arrests at the S + G_2_/M phases, they induced distinct cell distribution profiles where IR caused more intra-S phase arrest, while DCQ induced more G_2_/M arrest suggesting differences in their mechanisms of action. The combination treatment of DCQ+IR resulted in a strong arrest at 4 h where 61% of the population accumulated in the S+G_2_/M phases. Moreover, a significant increase in cell death represented by the sub-G_1 _population was associated with DCQ+IR (12.1% at 4 h versus 2.5% in non-treated cells). Even at early time-points this cytotoxic effect of the combination treatment appears to be at least additive. These results corroborate previous results that DCQ is anti-proliferative. We hypothesize that DCQ and IR act via different mechanisms. DCQ may cause DNA damage such as double-strand breaks (DSBs) or bulky adducts, which are known to induce S and G_2_/M arrest [14].

### DCQ Induces DNA Damage in EMT-6 Cells

**Figure 2:**
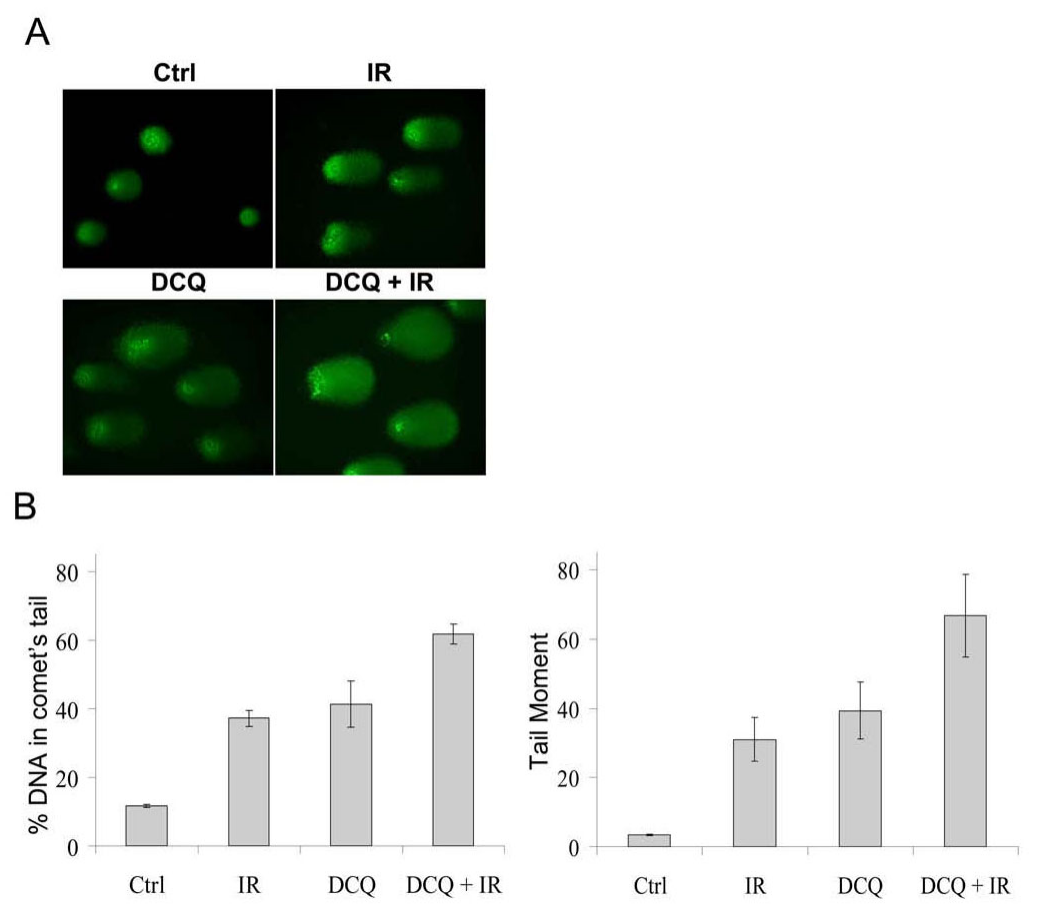
**DNA damage induced by DCQ+IR in EMT-6 cells**. **A**. Representative images of comets induced by DCQ± IR in EMT-6 cells subjected to the alkaline comet assay. EMT-6 cells were treated with IR (10 Gy), 4 h of DCQ (10 μM), or in combination. Cells were directly collected after the treatment and subjected to the alkaline comet assay. Images were taken using a fluorescent microscope at 40× (oil immersion) magnification. The tail lengths of the comets observed by each treatment are proportional to the amount of DNA damage induced. **B**. The mean of the parameters (% DNA in comet's tail and tail moment) are shown in the graphs above. The histograms summarize the averages of two independent experiments ± SE and show the mean of the %DNA in comet's tail and tail moments. More than 50 cells per treatment were photographed and quantified using TriTek CometScore software. SE: standard error.

Since DNA damage is the primary cause of arrest at S or G_2_/M phases, we tested whether DCQ induces DSBs in EMT-6 cells by using neutral comet assay. Although both treatments were observed to induce DSBs, the fluorescence intensity was too low to detect significant difference in the level of DSBs between DCQ and IR treatments (data not shown). The alkaline comet assay detects SSBs and alkaline-labile DNA damage, such as abasic sites. Using the alkaline comet assay, we detected the level of damage induced by DCQ ± IR in exponentially growing EMT-6 (Figure 2A, B). Cells were treated at 50% confluency with 10 μM DCQ and the assay was directly performed after a 4 h-incubation with DCQ, IR treatment, or combination treatment. Treatment with DCQ alone induced significant levels of damage, similar to that induced by 10 Gy IR. In response to combined DCQ and IR treatment, higher levels of damage were observed: tail moment (%DNA in tail × tail length) increased by 19.6-fold in comparison with untreated cells.

### DCQ Activates ATM and DNA-PK in Irradiated EMT-6 Cells

**Figure 3:**
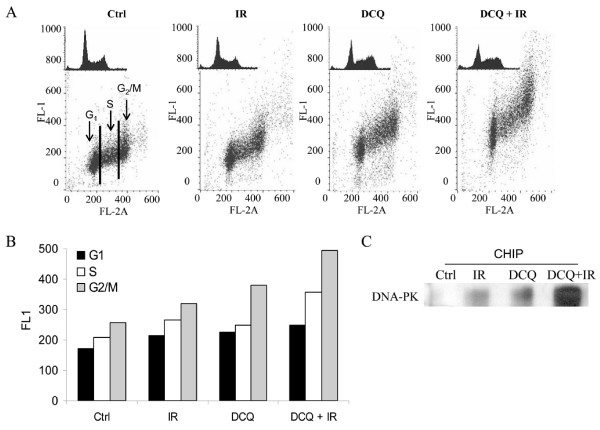
**Phosphorylation of ATM and activation of DNA-PK by DCQ ± IR in EMT-6 cells at 2 h post-treatment**. **A**. EMT-6 cells were treated with 10 μM DCQ, 10 Gy IR, or combination treatments, fixed and subjected to immunocytochemical detection of ATM phosphorylated on Ser1981, and stained with PI to detect at the same time p-ATM in each phase of the cell cycle. **B**. The mean of the FL-1 intensity (reflecting the level of p-ATM expression) at each phase of the cell cycle are plotted. **C**. Anti-DNA-PK was immunoprecipitated with DNA from lysates of 10^6 ^EMT-6 cells treated with 10 μM DCQ, 10 Gy IR, or combination treatments using CHIP assay. The immunoprecipitate was resolved on a 5% gel by electrophoresis, transferred to nitrocellulose and probed with anti-DNA-PK. The bands were quantified using LabWorks 4.0 software. CHIP: chromatin immunoprecipitation.

The nuclear kinase ATM is rapidly phosphorylated in the presence of low levels of DSBs [15]. The immunocytochemical detection of p-ATM thus provides a sensitive approach to detect double-strand breaks (DSBs) generated following drug treatment in cells [16]. Cells were treated with DCQ (10 μM), IR (10 Gy) or combinations followed by replenishment with drug-free media. After 2 h, cells were collected and the level of p-ATM in relation to the cell cycle was assayed in EMT-6 cells for each treatment by subjecting the samples to immunocytochemistry (Figure 3A, B). As expected, control cells showed the basal level of p-ATM expression was higher in G_2_/M population due to the role of ATM in mitosis. Exposure of EMT-6 cells to 10 μM DCQ triggered the activation of ATM by phosphorylation at Ser-1981; this phosphorylation level was higher than that of 10 Gy-treated cells, reflecting higher amounts of DSBs generated by DCQ than IR. The combination treatment had the highest levels of p-ATM over untreated cells reaching 2 fold only in the G_2_/M phase. DCQ and DCQ+IR induced the activation of ATM in all phases of the cell cycle similar to the Topoisomerase II inhibitor mitoxantrone [17].

Another major kinase activated by DNA damage is DNA-PK, which is activated by binding to the damaged sites on DNA [18]. The binding of DNA-PK to DNA was evaluated by DNA-PK chromatin immunoprecipitation followed by western blotting with an antibody against DNA-PK. We observed that untreated cells had no significant DNA-PK bound to the DNA, but a moderate signal was detected in EMT-6 cells after 10 μM DCQ or 10 Gy IR, and a highly significant increase in the active DNA-PK level was induced in response to DCQ+IR (Figure 3C).

### Slow Repair of Damage Observed in EMT-6 Exposed to DCQ+IR

**Figure 4:**
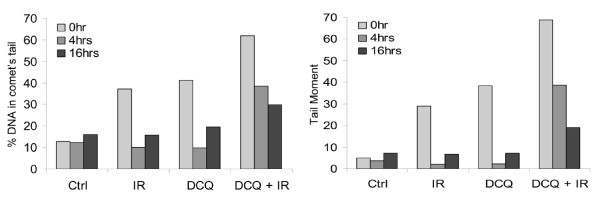
**Slow repair of DNA damage in DCQ+IR-treated cells**. Treated cells were either collected directly after treatment (0 h) or refreshed with drug-free media and incubated for another 4 or 16 h. The mean of the parameters (% DNA in comet's tail and tail moment) are shown in the graphs above.

The time required to repair DNA depends on the type of damage. SSBs are usually repaired much faster than DSB after induction [19]. To assess whether DCQ toxicity is due to the extent of damage induced or to slow repair following treatment, the extent of damage was assessed by the alkaline comet assay at 0 h, 4 h and 16 h post-treatments. Although a large extent of the damage induced by IR alone and DCQ alone is repaired in less than 4 h, we observed dramatically slowed repair of the damage induced by DCQ+IR. Even at 16 h, significant DNA damage remained unrepaired as evidenced by tail moments. Damage was significantly higher (P-value < 0.01) in response to DCQ+IR as compared to untreated and singly-treated cells (Figure 4).

### DCQ Generates Reactive Oxygen Species in EMT-6 cells

**Figure 5:**
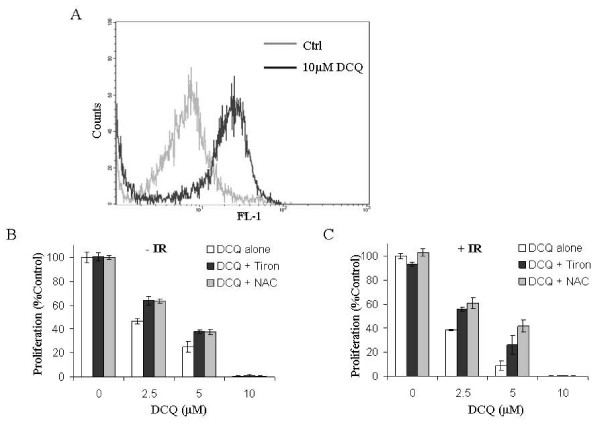
**ROS generation by DCQ in EMT-6 cells**. **A**. Cells were treated with 10 μM DCQ for 30 min before measuring ROS release by the DCFH-DA assay as described in the materials and methods. **B & C**. EMT-6 cells were pretreated for 2 h with the strong anti-oxidants Tiron and NAC, then with DCQ (2.5, 5 and 10 μM), and later subjected to 0 Gy (-IR) or 10 Gy (+IR) radiation. Afterwards, cells were replenished with drug-free media and incubated for an additional 20 h before the determination of cell proliferation. The values plotted represent an average (± SD) of two independent experiments. ROS: Reactive oxygen species.

N-oxides undergo redox-cycling producing reactive oxygen species (ROS) [20]. We hypothesized that DCQ may cause DNA damage by ROS induction due to its redox-cycling. Indeed, DCQ treatment alone, either directly or indirectly, induced the generation of ROS in EMT-6 cells after 30 min of treatment as measured by the DCFH assay (Figure 5A). To determine if ROS play a role in the radiosensitizing effect of DCQ in EMT-6 cells, strong anti-oxidants such as Tiron and NAC were added before treatment with DCQ alone or in combination with IR, to scavenge any DCQ-generated ROS. Cells pretreated with anti-oxidants were more resistant to the anti-proliferative effect of DCQ. However, the anti-oxidants did not completely abolish the anti-neoplastic effect of DCQ whether alone or in combination with IR (Figure 5B, C). These results indicate that ROS play at least a partial role in the radiosensitizing effect of DCQ in EMT-6 cells.

### DCQ Targets Rapidly-Proliferating Cells

**Figure 6:**
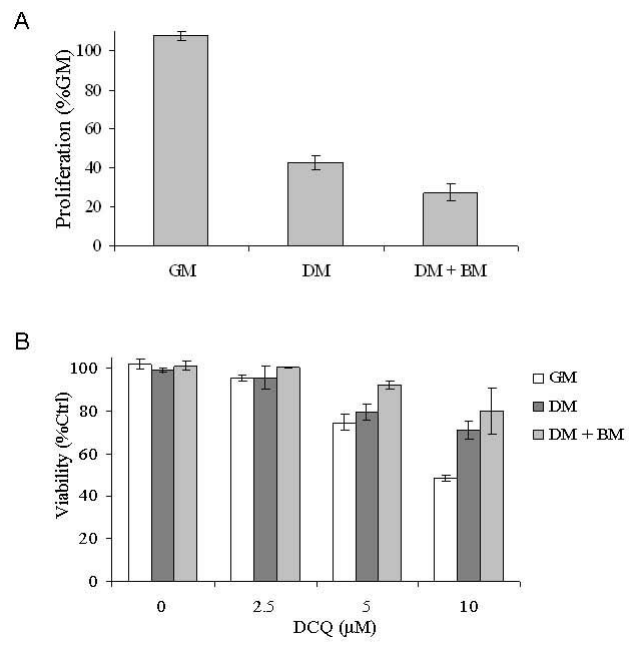
**Resistance of slow proliferating cells to DCQ**. **A**. After plating SCp2 cells in growth media (GM), they were subjected to different conditions. Cells were either kept in GM, or shifted 12 h after plating to differentiation media (DM) or DM+BM (basement membrane) for a more differentiated state. SCp2 cells were incubated for one day then assayed for proliferation using Cell Titer 96 non-radioactive cell proliferation. The proliferation rate of cells grown in GM was considered as 100%. **B**. The viability of SCp2 cells at different differentiating states were measured by Cytotox 96 non-radioactive cytotoxicity assay directly after 4 h of DCQ treatment.

Because DCQ appears to slow repair, we expected that toxicity would depend on proliferation rate. We assessed whether reducing proliferation would decrease DCQ toxicity by culturing murine mammary epithelial cell line SCp2 under conditions to induce differentiation and thereby slow proliferation. When cultured in differentiation media, SCp2 proliferation rate was reduced to approximately 50% compared to cells cultured in normal growth media, and an even stronger decrease in proliferation was observed with cells supplemented with basement membrane (Figure 6A). After 4 h of DCQ treatment, slowly proliferating SCp2 cells were more resistant to toxic concentrations of 10 μM DCQ, suggesting selective toxicity to proliferating cells (Figure 6B).

## Discussion

Several mechanisms of radiosensitization are known, including redox modulators [21], inhibitors of DNA damage repair [22], and regulators of growth factor receptors and other signaling molecules [23,24]. Misrepair of DNA damage causes mutation, and extensive damage may cause cell cycle arrest, or death if irreparable or too slowly repaired. The role ROS can play in cellular response to radiation has been well established [25].

Here, we show for the first time that DCQ induces DSBs in EMT-6 cells, in addition to SSBs and alkaline-labile lesions detected by the alkaline comet assay. DCQ causes more G2/M arrest than IR. Exposure of EMT-6 cells to 10 μM DCQ produced damage detected by the alkaline comet assay, and DSBs evaluated by p-ATM level, almost equivalent to that produced by 10 Gy IR. The combination of DCQ+IR induced significantly higher SSBs than each treatment alone. Radiosensitization of DCQ not only correlates with higher induction of DNA damage, but also with slower repair of this damage. Alkaline comet assays 4-hours post treatment revealed dramatically slowed repair of damage in DCQ+IR treated cells compared to separate IR or DCQ treatments. Little damage remained 4 h after separate treatments with DCQ or IR, supporting a model in which radiosensitization involves the generation of more difficult-to-repair DSBs. These results suggest combination treatment may have therapeutic value.

DNA damage, in particular DSBs, imposes a critical threat to the survival of cells if left unrepaired [26]. As a response to the damage, cells activate the DNA damage checkpoint. DSBs are detected by two main players in the DNA damage checkpoint: ATM and DNA-PK. Signal transduction, induced by the activation of these two signals, can cause cell-cycle arrest, repair, and cell death. Moreover, both are activated at very early stages of the DNA damage response, and are involved in DNA repair [27]. DNA-PK was activated in response to DCQ alone more than IR alone. The combination treatment induced the highest amount of active DNA-PK. ATM plays a critical role in S and G_2_/M phase arrest. Activated by DSBs, ATM becomes phosphorylated at Ser-1981 [15]. We show that ATM was activated in all phases of the cell cycle in response to the damage induced by all treatments. In the combination treatment the expression of p-ATM in G_2_/M phase was twice that of untreated cells.

Following IR treatment, EMT-6 cells arrest in S phase. Such an arrest is mainly caused by the activation of the intra-S-phase checkpoint due to significant amount of DSBs [28]. It is responsible for inhibition of DNA replication at late origins of replication. In addition to cell cycle arrest, the intra-S-phase checkpoint induces a cascade of reactions, that either attempt to repair the damage, mainly by homologous recombination, or induce cell death, depending on the extent of the damage induced. If damage is not repaired before the end of the S phase, cells would arrest at the G_2_/M DNA-damage checkpoint [28]. The G_2_/M arrest induced by DCQ and the S-phase accumulation induced by IR appear together in combination treatments. Despite our intriguing findings that the combination treatment DCQ+IR induces DNA damage, including DSBs, and slows repair, the precise mechanisms are still not clear.

The slow repair of DNA damage caused by DCQ+IR may have multiple contributing factors. DCQ appears to cause more DSBs than IR, as evidenced by the increase in p-ATM and DNA-PK levels. DCQ could create DSBs by generating closely opposed SSBs via ROS. Our observation of ROS generation upon DCQ treatment and the decrease in the sensitivity of cells to DCQ upon addition of anti-oxidants, support a role for redox cycling of DCQ. Although, the ROS scavengers did not completely reverse the effect of DCQ alone or in combination with IR, this does not eliminate the possibility that the radiosensitizing effect of DCQ may only involve ROS, because they may be mainly short-lived hydroxyl radicals that are not quenched by the anti-oxidants.

One possible mechanism of DCQ radiosensitization is that IR induces a higher concentration of the free radical character of DCQ, which is translated into increased SSBs and DSBs. The increased levels of SSBs, in combination with increased levels of difficult-to-repair DSBs could overwhelm cellular DNA repair pathways. The proposed mechanism was observed to be selective for rapidly proliferating cells, presumably because slowly dividing cells have more time to repair DNA damage. This finding suggests clinical potential.

## Conclusion

This study presents evidence that the radiosensitizing effects of DCQ are associated with an increase in DNA damage, including DSBs, the activation of the key DNA damage markers, p-ATM and DNA-PK, and the generation of ROS. The significant levels of unrepaired damage detected by alkaline comet assay in EMT-6 cells following treatment by DCQ+IR indicate that decreased DNA repair contributes to the mechanism of DCQ radiosensitization.

## Competing interests

The authors declare that they have no competing interests.

## Authors' contributions

JH carried out the experiments in the study and drafted the manuscript. FG and CAS were involved in revising the manuscript critically for important intellectual content and CAS helped in preparing the final draft of the manuscript. MH provided the compound and reviewed the manuscript. HGM conceived of the study, and participated in its design and coordination and drafting of the manuscript. All authors read and approved the final manuscript.
